# Prevalence and genetic diversity of *Trichomonas vaginalis* clinical isolates in a targeted population in Xinxiang City, Henan Province, China

**DOI:** 10.1186/s13071-018-2753-4

**Published:** 2018-03-02

**Authors:** Zhenchao Zhang, Lixia Kang, Weijuan Wang, Xin Zhao, Yuhua Li, Qing Xie, Shuai Wang, Tong He, Han Li, Tingwei Xiao, Yunchao Chen, Suqiong Zuo, Lingmin Kong, Pengju Li, Xiangrui Li

**Affiliations:** 1 School of Basic Medical Sciences, Xinxiang Medical University, Xinxiang, Henan 453003 People’s Republic of China; 2 The Third Affiliated Hospital of Xinxiang Medical University, Xinxiang, Henan 453003 People’s Republic of China; 3 The First Affiliated Hospital of Xinxiang Medical University, Xinxiang, Henan 453003 People’s Republic of China; 4 College of Veterinary Medicine, Nanjing Agricultural University, Nanjing, Jiangsu 210095 People’s Republic of China

**Keywords:** *Trichomonas vaginalis*, Prevalence, Genetic diversity, China

## Abstract

**Background:**

*Trichomonas vaginalis* (TV) is a protozoan parasite that causes trichomoniasis, a sexually transmitted disease, worldwide. In this study, we investigated the prevalence and genetic characterization of *T. vaginalis* and contrasted the most prevalent strains of *T. vaginalis* isolated from Xinxiang City, Henan Province, China.

**Results:**

In Xinxiang from September 2015 to September 2017, a total of 267 (1.64%, 95% confidence interval, CI: 1.45–1.85) clinical *T. vaginalis*-positive samples from vaginal secretions were observed by wet mount microscopy from 16,294 women with some clinical symptoms of trichomoniasis. We found that trichomoniasis frequently occurred in the 21- to 40-year-old age group and in winter. After the 267 clinical *T. vaginalis* positive samples were cultured, 68 isolates of *T. vaginalis* were harvested and identified as genotype E (58.82%), H (17.65%), mixed 1 (17.65%) and mixed 2 (5.88%) using a sensitive and reliable polymerase chain reaction-restriction fragment length polymorphism (PCR-RFLP) typing method on the actin gene. The phylogenetic diversity analysis showed that the genotype E samples fell within a separate clade compared to the other *T. vaginalis* isolates, while the samples of the genotype H separated into two clades.

**Conclusions:**

Our results demonstrate a notable gene polymorphism of clinical isolates from the targeted population and provide insight into the performance of these genetic markers in the molecular epidemiology of trichomoniasis. However, further studies are needed to clarify the association between a certain genotype and the pathogenicity of *T. vaginalis*.

## Background

*Trichomonas vaginalis* (TV), which causes non-viral sexually transmitted disease, is a protozoan that parasitizes the urogenital tract of humans, leading to trichomoniasis. The World Health Organization (WHO) estimated that 248 million people were infected with TV in 2005, and by 2008, this number had increased by 11.2% to 276.4 million [1]. The pathogen infects not only women but also men, and the infection is frequently asymptomatic in both women and men. In women, the disease may range from the absence of symptoms (in up to 50% of women) to severe vaginitis and urethritis symptoms, and patients infected with TV are at high risk of cervical cancer and adverse pregnancy outcomes [2]. Although men are often asymptomatic carriers of *T. vaginalis*, dysuria and discharge have also been reported, and *T. vaginalis* infection also increases the risk of infertility and prostate cancer in men [3]. Moreover, the infection of *T. vaginalis* has been linked with increased risk and transmission of HIV infection [4].

Trichomoniasis is primarily treated with drugs; metronidazole is a common drug used to treat this disease [5]. However, the Centers for Disease Control and Prevention (CDC) has predicted that 2–5% of clinical *T. vaginalis* isolates have some level of drug resistance to metronidazole [6].

Despite the public health importance and global distribution of trichomoniasis, we have little knowledge of the various features of the infection of *T. vaginalis*. Moreover, the pathogenicity, drug resistance and other epidemiological aspects of *T. vaginalis* have still not been clarified. Thus, studies based on the genetic analysis of the parasite would be useful. PCR and its related methods are widely used in molecular epidemiology for genetic studies of organisms, depending on their sensitivity and reliability [7]. Thus far, these methods for studying the genetic diversity of parasites include PCR-hybridization, PCR-size polymorphism, random amplification of polymorphic DNA (RAPD) and PCR-RFLP [8]. PCR-RFLP possesses both the reliability of RFLP and the sensitivity of PCR; therefore, it is an appropriate method of strain typing assessment [8].

With approximately 60,000 protein-coding genes, the genome of *T. vaginalis* is 160 Mb. Many repeated and transposable elements are contained in the genome that can vary in position, and the genome may explain the noticeable genetic variability of *T. vaginalis* [9]. As the structural proteins present in the eukaryotic cytoskeleton, the protein of actin is encoded by a gene family that includes no fewer than nine members in *T. vaginalis* [10]. Morphological changes of *T. vaginalis*, such as surface adhesion and the form alternation from flagellate to amoeboid, are considered to be influenced by actin. In addition, the actin protein has a conserved nature of ubiquity [11], which makes its use feasible for identifying intra-species molecular diversity.

Until now, studies of the genetic diversity and molecular epidemiology of *T. vaginalis* were rare in China. To obtain more knowledge regarding the genetic properties of *T. vaginalis* in Xinxiang City, Henan Province, China, based on the actin gene, we assessed the situation of genetic diversity in *T. vaginalis* isolates through PCR-RFLP and sequence analysis.

## Methods

### *Trichomonas vaginalis* isolates

From September 2015 to September 2017, vaginal secretions were collected by medical staff through swabs from 16,294 women with some clinical symptoms of trichomoniasis at the Third Affiliated Hospital of Xinxiang Medical University. These samples were examined by wet mount microscopy, and the positive samples of clinical *T. vaginalis* were cultured on TYM medium with antibiotics (50 mg/ml ciprofloxacin, 100 mg/ml ceftriaxone), fungicides (2.5 mg/ml amphotericin B) and 10% calf serum at a temperature of 37 °C. We harvested the parasites by centrifugation at the stationary phase (2 × 10^6^ parasites) and applied DNA extraction directly.

### DNA extraction

We washed the trophozoite of *T. vaginalis* three times in PBS (pH 7.2) at 5000 rpm for 5 min and then extracted the nucleic acids with E.Z.N.A.® Tissue DNA Kit (OMEGA, Zhengzhou, China), in accordance with the manufacturer’s instructions.

### PCR-RFLP

We chose the target of the nested PCR based on the actin gene (GenBank: AF237734). The selection of outer primers (OPs) and inner primers (IPs) was done according to references [12, 13], and the primer sequences are provided in Table 1. The length of the target was 1100 bp. The PCR amplification was conducted using a thermocycler (Bio-Rad, USA) in two steps. The first step of the PCR reaction was composed of 5 μl DNA, 12.5 μl master mix (Yi Fei Xue Biotechnology, Nanjing, China), and 2 μl OPs (20 pmol each of OP-F and OP-R) in 25 μl of final volume. The second step of the PCR reaction was composed of 5 μl first stage PCR products, 25 μl master mix, and 4 μl IPs (20 pmol each of IP-F and IP-R) in 50 μl of final volume. There were 35 cycles in the first step. Each cycle was composed of denaturation (95 °C for 45 s), annealing (55 °C for 30 s), and extension (72 °C for 1 min). Denaturation at 95 °C was conducted for 5 min before the first cycle, and a 10 min final extension at 72 °C was conducted after the last cycle. There were also 35 cycles in the second step. The denaturation and extension were the same as those in the first step, but the temperature for annealing was 50 °C. After PCR, we analyzed 5 μl PCR product through electrophoresis on 1% agarose gel in Tris-acetate-EDTA buffer (TAE, pH 8.5) and then visualized it under UV light with 0.5 μg/ml ethidium bromide staining (Applichem, Biochemica, Darmstadt, Germany). The length of the target was 1100 bp, which is only 28 bp shorter than the size of the actin gene’s open reading frame.

**Table 1:** Oligonucleotide primer sequences used for Nested PCR in this research

Name	Sequences (5'-3')	Description
OP-F	TCTGGAATGGCTGAAGAAGACG	Forward primer for the actin gene of *T. vaginalis* in the first stage
OP-R	CAGGGTACATCGTATTGGTC	Reverse primer for the actin gene of *T. vaginalis* in the first stage
IP-F	CAGACACTCGTTATCG	Forward primer for the actin gene of *T. vaginalis* in the second stage
IP-R	CGGTGAACGATGGATG	Reverse primer for the actin gene of *T. vaginalis* in the second stage

For RFLP, we digested 10 μl amplified product using 0.5 μl of each restriction endonuclease *Hin*dII (10 U/μl, 500 units), *Mse*I (10 U/μl, 300 units) and *Rsa*I (10 U/μl, 1000 units), separately (Yi Fei Xue Biotechnology, Nanjing, China). We incubated the reaction at 37 °C for 4 h. We separated the fragments on a 3% agarose gel in TAE buffer, as previously described. Ultimately, we stained the gel using ethidium bromide and visualized it with a transilluminator. We measured the size of the amplified products with a 100 bp commercial weight marker (Yi Fei Xue Biotechnology, Nanjing, China).

### Sequence analysis

All clinical isolates were sequenced using the Sanger technique (Genewiz, Suzhou, China) for determination of the fragments size and confirmation of the banding patterns. We edited and aligned the sequences using Clustal W (http://www.ebi.ac.uk/Tools/msa/clustalo/) and compared them with the reference sequences of GenBank (EU076580, AF237734, EU076582, EU076584, EU076579, EU076578, EU076585, EU076583, EU076586 and AB468096). For the analysis of phylogenetic diversity of *T. vaginalis*, we built a phylogenetic tree with molecular evolutionary genetics analysis (MEGA) software (version 6.0) [14] using the neighbor-joining (NJ) algorithm, containing sequences representing *T. vaginalis* isolates from GenBank.

### Statistical analysis

We performed all statistical analyses with the SPSS 20 software for Windows (SPSS Inc, Chicago, IL, USA). The Chi-square test was used for statistical analyses of the prevalence of *T. vaginalis* under the different variables, and *P* < 0.05 was considered to indicate a statistically significant difference.

## Results

### The prevalence of *T. vaginalis*

A total of 267 (1.64%, 95% CI: 1.45–1.85%) clinically positive *T. vaginalis* samples from vaginal secretions were collected from 16,294 women with some clinical symptoms of trichomoniasis, including vaginitis, urethritis, low birth weight infants and preterm delivery, premature rupture of membranes, and infertility. After the 267 clinically positive *T. vaginalis* samples were cultured on TYM medium with antibiotics, fungicides and 10% calf serum at 37 °C, 68 isolates of *T. vaginalis* were harvested and observed through a microscope.

**Fig. 1:**
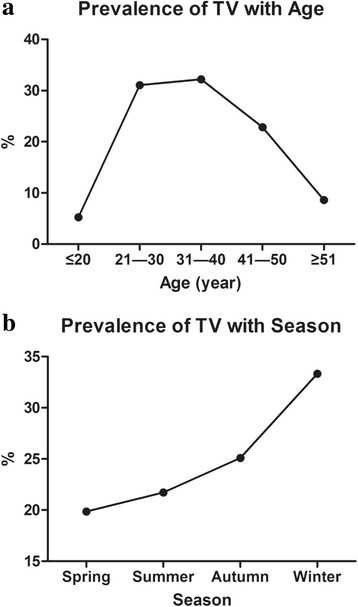
Epidemic characteristics of trichomoniasis by age and season in Xinxiang. **a** Prevalence of *T. vaginalis* in different age groups. **b** Prevalence of *T. vaginalis* in the four seasons of the year

In the present study, as shown in Fig. 1a and Table 2, a significant difference was observed in the prevalence of *T. vaginalis* in different age groups (*χ*^2^ = 104.71, *df* = 4, *P* < 0.001). In the 267 women infected with *T. vaginalis*, higher infection rates were detected in 21- to 30-year-old women (31.09%, *n* = 83) and 31- to 40-year-old women (32.21%, *n* = 86), followed by the infection rate (22.85%, *n* = 61) in 41- to 50-year-old women, while the infection rates found in women ≤ 20 years of age and ≥ 51 years of age were 5.24% (*n* = 14) and 8.61% (*n* = 23), respectively. In the ≤ 30 years of age group, the infection rate of *T. vaginalis* in women increased significantly (*χ*^2^ = 59.98, *df* = 1, *P* < 0.001) following age increase, while in the ≥ 31 years of age group, the infection rate decreased significantly (*χ*^2^ = 45.09, *df* = 2, *P* < 0.001) following age increase. The infection rate of *T. vaginalis* in women was highest (63.30%, *n* = 169) in the 21- to 40-year-old age group.

**Table 2:** The prevalence of *T. vaginalis* in women in Xinxiang City, Henan Province, China

Variable	No. of infections	Infection rate (%)
Age (years)		
≤ 20	14	5.24^a^
21–30	83	31.09^b^
31–40	86	32.21^b^
41–50	61	22.85^c^
≥ 51	23	8.61^a^
Season		
Spring	53	19.85^a^
Summer	58	21.72^a^
Autumn	67	25.09^a^
Winter	89	33.33^b^
Total	267	100

*Note*: Values bearing a different superscript letter (^a-c^) within a column differ significantly from one another (*P* < 0.05)

In addition, a significant difference was observed in the prevalence of *T. vaginalis* in the four seasons of the year (*χ*^2^ = 15.20, *df* = 3, *P* = 0.002) (Fig. 1b, Table 2). In the 267 women infected with *T. vaginalis*, 19.85% (*n* = 53) and 21.72% (*n* = 58) of infections were found in the spring and summer, respectively, and 25.09% (*n* = 67) was found in autumn, while the infection rate found in women in winter was 33.33% (*n* = 89). Thus, the infection rate of *T. vaginalis* in women was highest in winter in the city of Xinxiang.

### Nested PCR

The amplification of the actin gene of *T. vaginalis* by nested PCR demonstrated a 1100 bp fragment among all isolates, and agarose gel electrophoresis indicated that there was no difference in the length of the 68 amplicons.

### *Trichomonas vaginalis* actin genotypes

The actin genotypes, length of DNA fragments and pattern groups of the *T. vaginalis* isolates are demonstrated in Table 3. The restriction enzyme *Hin*dII digested the amplified products into two patterns: four distinct DNA fragments of 60, 213, 401 and 426 bp or three DNA fragments of 60, 213 and 827 bp. Digestion of the amplified product using *Rsa*I produced four patterns, including six distinct DNA fragments of 87, 103, 106, 116, 236 and 452 bp; five DNA fragments of 106, 116, 190, 236 and 452 bp; five DNA fragments of 87, 103, 106, 236 and 568 bp; and four DNA fragments of 106, 190, 236 and 568 bp. Digestion of the amplified product using *Mse*I produced three patterns, including: three distinct fragments of 186, 333 and 581 bp and two DNA fragments of 519 and 581 bp.

**Table 3:** Size of fragments, pattern groups and actin genotypes of the *T. vaginalis* (extracted from [12])

Genotype	Restriction with *Hin*dII (bp)	Restriction with *Rsa*I (bp)	Restriction with *Mse*I (bp)
A	827, 213, 60	568, 236, 190, 106	581, 519
E	827, 213, 60	568, 236, 106, 103, 87	581, 315, 204
G	426, 401, 213, 60	568, 236, 190, 106	581, 519
H	426, 401, 213, 60	568, 236, 106, 103, 87	581, 519
I	426, 401, 213, 60	452, 236, 190, 116, 106	581, 519
M	426, 401, 213, 60	568, 236, 190, 106	581, 333, 186
N	426, 401, 213, 60	568, 236, 106, 103, 87	581, 333, 186
P	426, 401, 213, 60	452, 236, 116, 106, 103, 87	581, 333, 186

**Fig. 2:**
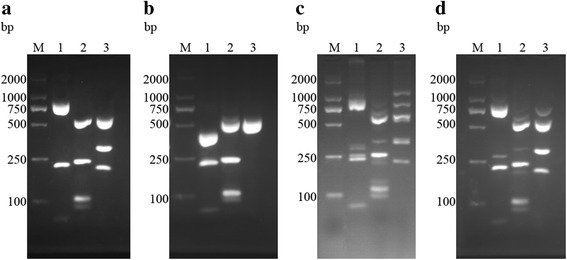
DNA fragment patterns of isolates after the digestion of actin genotypes E (**a**), H (**b**), mixed 1 (**c**) and mixed 2 (**d**) on 3% agarose gel. Lane M: 2000 bp DNA marker; Lane 1: banding patterns after digestion with *Hin*dII; Lane 2: banding patterns after digestion with *Rsa*I; Lane 3: banding patterns after digestion with *Mse*I

**Fig. 3:**
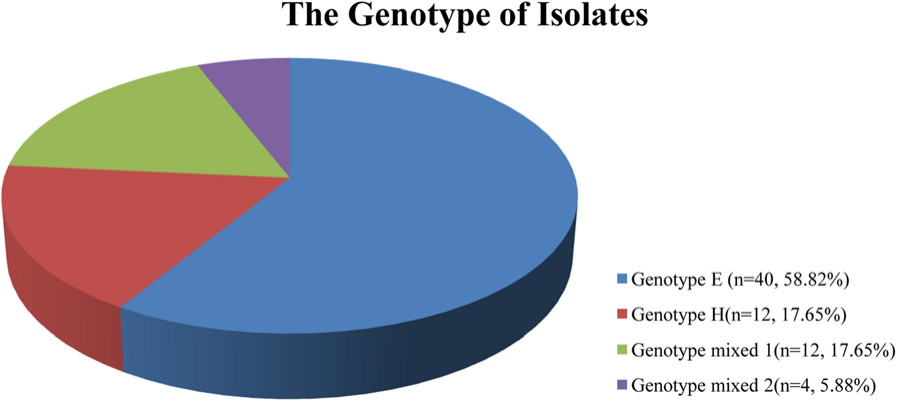
Percentage of actin genotypes E, H, mixed 1 and mixed 2 of the isolates

Separation of the DNA fragments after gel electrophoresis is shown in Fig. 2. The figure demonstrates the patterns of DNA fragments of isolates 14, 19, 42 and 56, which display actin genotypes E, H, mixed 1 and mixed 2, respectively. As shown in Fig. 3, the major genotype was type E (58.82%), followed by type H (17.65%), type mixed 1 (17.65%), and type mixed 2 (5.88%). In addition, the actin genotypes of A, G, I, M, N and P were not found in these isolates.

### Phylogenetic diversity

In addition to the samples of type mixed, 52 sequences of the actin gene from the remaining *T. vaginalis* isolates were obtained and analyzed. In these sequences, there were 2 different sequences in genotype H and 3 different sequences in genotype E. The 5 sequences were submitted to GenBank under the accession numbers MG253641, MG253642, MG253643, MG253644 and MG253645.

**Fig. 4:**
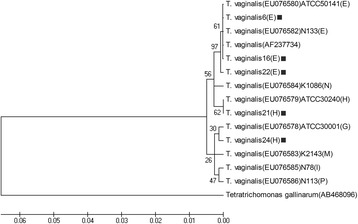
A phylogenetic tree of Xinxiang and reference *trichomonad* isolates, based on the actin gene. The five sequence types identified in this study are shown with a dark square

To assess genetic diversity among the *T. vaginalis* isolates, we conducted a multiple alignment of the various *T. vaginalis* actin genotypes with the sequences found in this study and reference isolates and constructed a tree with the NJ algorithm (Fig. 4). The five sequences detected in the present study are shown with a dark square in this phylogenetic tree. We drew branch lengths to scale, according to genetic distances between isolates, and this figure demonstrates that *T. vaginalis* 24 (H) is heterogeneous and not located in the cluster of type H. The phylogenetic diversity analysis showed that the genotype E samples fell within a separate clade compared to the other *T. vaginalis* isolates, while the samples of the genotype H separated into two clades.

## Discussion

Trichomoniasis is a common sexually transmitted disease. Between 2011 and 2014, the prevalence of *T. vaginalis* was 2.4 % in Granada, Southern Spain [15]. In Korea, a cross-sectional analysis was implemented to investigate the prevalence of sexually transmitted infections triggered by the pathogens *T. vaginalis*, *Neisseria gonorrhoeae*, human immunodeficiency virus (HIV), herpes simplex virus (HSV), *Mycoplasma genitalium*, *Chlamydia trachomatis*, *Mycoplasma hominis*, *Ureaplasma parvum*, *Treponema pallidum* and *Ureaplasma urealyticum*, and among them, the prevalence of *T. vaginalis* was 0.8% [16]. In Western Canada, the prevalence of *T. vaginalis* (2.8%) in female sexually transmitted infection clinic attendees was within the prevalence of gonorrhea (1.8%) and chlamydia (5.8%), while the prevalence of *T. vaginalis* was low for male attendees (0.2%) [3]. Moreover, a cross-sectional study was conducted in a city of Yunnan Province in southern China, and the prevalence of *T. vaginalis* was 9.0% in 734 female sex workers [17]. In the present study, 16,294 vaginal secretions were collected from women with some clinical symptoms of trichomoniasis in the city of Xinxiang, Henan Province, China, and among them, 267 (1.64%, 95% CI: 1.45–1.85%) clinically positive *T. vaginalis* samples were observed by wet mount microscopy. In addition, our study showed that the infection rate of *T. vaginalis* was highest in women 21 to 40 years old, and the infection rate increased significantly (*χ*^2^ = 59.98, *df* = 1, *P* < 0.001) as age increased in the ≤ 30 years of age group and decreased significantly (*χ*^2^ = 45.09, *df* = 2, *P* < 0.001) as age increased in the ≥ 31 years of age group. The infection rate of *T. vaginalis* in winter was higher than that in other seasons. The diagnostic method of wet mount microscopy for *T. vaginalis* has been shown to have low sensitivity. Thus, the result of infection rate of *T. vaginalis* might be influenced by this diagnostic method.

Because of the lack of awareness about the genetic distribution and mapping of parasites, there have been few studies on *T. vaginalis* transmission routes, virulence factors and the prevalence and sensitivity to metronidazole of various genotypes [18]. The technique of strain typing is important for investigating the epidemiology of parasitic infections. Thus far, the techniques of molecular typing for *T. vaginalis* include multilocus sequence typing (MLST), RAPD-PCR, PCR-RFLP and sequencing polymorphism based on multifarious markers, for instance, ITS and microsatellite [12, 19, 20]. In these techniques, PCR-RFLP based on the actin gene was used for strain typing of *T. vaginalis* clinical isolates as a reproducible and sensitive tool. A promising study has been conducted through PCR-RFLP analysis of the actin gene with three restriction enzymes (*Mse*I, *Rsa*I and *Hind*II); the digestion patterns showed that 8 different genotypes were identified as the prominent genotypes in Zambia and Kinshasa, and the actin genotypes G and E were the predominant genotypes [12].

Moreover, in the isolates from Kerman and Shiraz, Tavakoli Oliaee et al [14] identified various genotypes of *T. vaginalis*, among which the H and I genotypes were the most frequent types, respectively. Although actin genotypes A and P of *T. vaginalis* have been found in previous studies, neither were identified in the targeted population [14]. In Kenya, five actin genotypes were revealed by RFLP analysis in 2015, and 50.0% of the isolates were of actin genotype E, 27.3% of actin genotype N, 13.6% of actin genotype G and 4.5% of actin genotypes I and P [21]. Based on *18S* rRNA gene sequences, genetic variants among *T. vaginalis* were found in the cities of Anyang, Zhengzhou, Shangqiu, Luoyang, Pingdingshan, Zhumadian and Xinyang, Henan Province, China, and the results indicated that the *T. vaginalis* isolates identified could be regarded as a single population [22]. In this study, two kinds of *T. vaginalis* actin genotypes were identified through PCR-RFLP, which targeted the actin gene. Various fragment patterns gained from RFLP have demonstrated that the types of E and H were confirmed through the designation of their nucleotide sequences, with frequencies of 58.82% and 17.65%, respectively. However, other actin genotypes of *T. vaginalis* (A, G, I, M, N and P) were not found in this study. In addition, the patterns of mixed 1 and mixed 2 genotypes were identified only in the city of Xinxiang, with frequencies of 17.65% and 5.88%, respectively. However, classification of mixed 1 and mixed 2 genotypes requires further study.

In this study, 2 different sequences and 3 different sequences were found in types H and E, respectively. The analysis of phylogenetic diversity showed that *T. vaginalis* 6, *T. vaginalis* 16 and *T. vaginalis* 22 belonged to genotype E, which was consistent with the result of PCR-RFLP. However, according to the phylogenetic diversity results, *T. vaginalis* 21 and *T. vaginalis* 24 were genotype H and G, respectively, which differed from the analysis of PCR-RFLP. Sequence alignment indicated that the “T” nucleotide was replaced by the “C” nucleotide (T→C) in the nucleic acid sequence sites 149, 914 and 917 of *T. vaginalis* 24. Thus, the nucleic acid sequence sites 149, 914 and 917 might be the key sites of the *T. vaginalis* gene polymorphism. Moreover, the genotyping technique for *T. vaginalis* needs further study.

Although *T. vaginalis* is recognized as having the feature of high-level diversity, this heterogenetic protozoan is a parasite with a unique population structure, including two different types with equivalent proportions worldwide [18]. In the present study, the result of phylogenetic diversity showed that the genotypes of *T. vaginalis* came originally from two different clades, and this finding was consistent with other studies [20, 23, 24]. Moreover, our study showed that the E genotype of *T. vaginalis*, as the major genotype, was present in the same clade, while the H genotype was derived from two distinct clades.

## Conclusions

Even though genetic variants of *T. vaginalis* have already been analyzed based on 18S rRNA in Henan Province [22], the content samples were limited, and the city of Xinxiang was not included. In this study, we found that 1.64% (95% CI: 1.45–1.85%) of women were observed to be infected by *T. vaginalis* according to wet mount microscopy of 16,294 women with some clinical symptoms of trichomoniasis in the city of Xinxiang, Henan Province, China, and the disease occurred frequently in the 21- to 40-year-old group and in winter. After being cultured, 68 isolates of *T. vaginalis* were harvested and identified as genotype E, H, mixed 1 or mixed 2 based on the actin gene. The phylogenetic diversity indicated that the genotypes of *T. vaginalis* were derived from two distinct clades. The findings provided an insight into evaluating the performance of these genetic markers in molecular epidemiology of trichomoniasis. However, more studies are needed to clarify the association between a certain genotype and the manifestation of a clinical infection.

## Abbreviations

CDC: Centers for Disease Control and Prevention
HIV: Human immunodeficiency virus
HSV: Herpes simplex virus
IPs: Inner primers
MLST: Multilocus sequence typing
PBS: Phosphate buffer saline OPs Outer primers
PCR-RFLP: Polymerase chain reaction - restriction fragment length polymorphism
RAPD: Random amplification of polymorphic DNA
TV: *Trichomonas vaginalis*
TYM: Trypticase yeast extract maltose
WHO: World Health Organization
